# Protective Effects of Vasodilatory Βeta-Blockers Carvedilol and Nebivolol against Glycerol Model of Rhabdomyolysis-Induced Acute Renal Failure in Rats

**DOI:** 10.3889/oamjms.2016.082

**Published:** 2016-08-01

**Authors:** Ahmed Atwa, Rehab Hegazy, Nermeen Shaffie, Neamat Yassin, Sanaa Kenawy

**Affiliations:** 1 *Egyptian Russian University, Badr City, Egypt*; 2 *Pharmacology Department, Medical Division, National Research Center, Giza, Egypt*; 3 *Pathology Department, Medical Division, National Research Center, Giza, Egypt*; 4 *Pharmacology and Toxicology Department, Faculty of Pharmacy, Cairo University, Cairo, Egypt*

**Keywords:** Rhabdomyolysis, Acute renal failure, Carvedilol, Nebivolol, Glycerol, NO, Rat

## Abstract

**BACKGROUND:**

Rhabdomyolysis (RM)-induced acute renal failure (ARF) accounts for about 10–40% of all cases of ARF.

**AIM:**

The present study investigated the possible protective effect of two nitric oxides (NO)-releasing third generation β-blockers, carvedilol (Carv) and nebivolol (Nebi), against RM-mimicking glycerol (Gly)-induced ARF in rats.

**MATERIAL AND METHODS:**

After 24 h dehydration, rats received a single dose of 50% Gly (8 ml/kg, im). They were treated with vehicle, Carv (2.5 mg/kg/day, po) or Nebi (10 mg/kg, po) for 3 successive days starting from an hour prior to Gly injection. Evaluation of blood pressure and locomotor activity was performed during the experiment. 72 h following Gly administration, total protein in the urine, serum levels of creatinine, blood urea nitrogen, sodium and potassium as well as the renal contents of malondialdehyde, reduced glutathione and NO were assessed, together with a histopathological examination of renal tissues.

**RESULTS:**

Carv and Nebi attenuated Gly-induced renal dysfunction and histopathological alterations. They decreased the Gly-induced oxidative stress and increased renal NO concentration. Restoration of normal blood pressure and improvement of locomotor activity were also observed.

**CONCLUSION:**

The results clearly demonstrate protective effects of Carv and Nebi against renal damage involved in RM-induced ARF and suggest a role of their antioxidant and NO-releasing properties.

## Introduction

Rhabdomyolysis (RM) is an important cause of acute renal failure (ARF). It results in about 10-40% of all cases [1]. The term rhabdomyolysis refers to the disintegration of skeletal muscles leading to the release of intracellular myoglobin (Mb), enzymes and electrolytes from myocytes into blood circulation [2]. It may be caused by trauma, ischemia, some drugs, toxins, metabolic disorders, or infections [3].

Many factors are known to contribute to RM-induced ARF; one of them is hypovolemia that results from the accumulation of a large amount of intravascular fluid in the space created from the damage of muscular tissue [4]. This hypovolemia resulted in a considerable reduction in renal blood flow (RBF) and glomerular filtration rate (GFR) that lead to ARF [5]. Hypovolemia is also associated with the sympathetic nervous system and reticular angiotensin aldosterone system (RAAS) activation with increased production of vasoconstricting molecules and inhibition of production of vasodilatory prostaglandins [6]. These, together with the vasoconstricting endotoxins and cytokines released into the systemic circulation after muscle damage, lead to renal hypoperfusion and tissue injury [7]. Another important factor contributes to RM-induced ARF is Mb that released by the dead myocytes. Mb scavenges nitric oxide (NO) which is the most potent endogenous vasodilatory factor, and this contributes to the renal hypoperfusion and tissue injury in the setting of RM [8]. In addition, the intracellular degradation of Mb at the urinary pH to globin and ferriheme leads to free iron overloading of tubular cells [8]. Free iron is an oxidative metal that either facilitates the production of oxygen free radicals or acts as a free radical by itself [9]. This oxidative stress generated in the cytoplasm of tubular cells increases oxidation of lipids, proteins and DNA that resulting in ARF [10]. Lipid peroxidation occurs in the kidney markedly increases isoprostanes which are also potent vasoconstrictors [11].

Carvedilol (Carv) is a third-generation, non-selective β-blocker that also possesses α_1_-adrenergic blocking activity [12]. It is indicated for the treatment of essential hypertension, heart failure, and post-myocardial infarction left ventricular dysfunction [13]. Data indicate that the vasodilation effect of Carv is mediated through both α_1_-adrenergic receptor blockade and enhanced endothelial NO release [14]. Carv also has a number of ancillary activities including antioxidant, anti-inflammatory, anti-apoptotic, anti-ischemic, anti-proliferative, and Ca^2+^ antagonist properties [15]. These properties may provide protection for several major organ systems including the heart, blood vessels, kidneys and brain [16]. Carv has been found to decrease renal vascular resistance and improve renal hemodynamics by improving RBF and GFR [17]. Other renoprotective effects of Carv were found to be independent of its vasodilatory effect rather than its antioxidant and antiproliferative properties as well as its capability to reduce expression of profibrotic factors [18].

Nebivolol (Nebi) is a third generation selective β_1_-adrenergic receptor blocker with vasodilator properties mediated by a direct stimulatory effect on the endothelial nitric oxide synthase (eNOS) (L-arginine-NO pathway). Nebi has also been shown to reduce the expression and protein levels of molecules involved in adhesion, inflammation, hypertension, and vascular remodelling that are induced by oxidative stress [20]. Treatment with Nebi has been shown to decrease renal fibrosis and glomerular injury as well as improving endothelial dysfunction. These effects have been attributed to vasodilatation, reduction in oxidative stress in addition to the enhancement of NO bioavailability [21].

Taken together, these pharmacological properties of both drugs, Carv and Nebi, with their renoprotective effects could be of potential interest in patients with renovascular diseases such as RM-induced ARF. For that, the present study was performed to investigate the possible protective effects of them against an RM-mimicking Gly-induced ARF in rats.

## Material and Methods

### Animals

Adult male Wistar rats weighing 150-200 g were utilised in the present study. Standard food pellets and tap water were supplied *ad libitum* unless otherwise stated. Animals and food pellets were obtained from the animal house colony of the National Research Center (NRC) (Cairo, Egypt). All the animal experiments were carried out in accordance with guidelines evaluated and approved by the ethics committee of NRC (Cairo, Egypt).

### Drugs

Carv and Nebi were obtained from Sigma-Aldrich (USA). They were available as a powder, and used in the current study at doses of 2.5 mg/kg, po [22] and 10 mg/kg, po [23], respectively. Drugs were freshly prepared at the beginning of each experiment by being suspended in distilled water and volumes were adjusted so each rat received 1 ml suspension/100 g body weight. All other chemicals used were of the highest purity available.

### Experimental Design

RM-induced ARF in rats was induced using a single dose of hypertonic glycerol (Gly) solution (50% v/v in sterile saline) following 24 h of dehydration [24]. Animals were randomly allocated into four groups; each group consisted of 10 rats. The rats received an injection of Gly solution (8 ml/kg, i.m.) or equal volume of saline for animals of the 1^st^ group, which served as the normal control. The injected volume was divided equally between the two hind limbs. Administration of drugs was carried out daily for 3 successive days, starting 60 min prior to the Gly injection. The first 2 groups, normal and RM-ARF groups, received saline orally, and the other 2 groups received Carv (2.5 mg/kg/d, po) and Nebi (10 mg/kg/d, po), respectively. Animals were allowed free access to food and tap water during the course of the experiment, while rats of the last 3 groups were deprived of drinking water for 24 h before the Gly administration.

### Assessment of locomotor activity

On day 0 and 1 h following the last drug administration, locomotor activity was measured by detecting rat movements using grid floor activity cage (Model no. 7430, Ugo-Basile, Italy). Interruptions of infrared beams were automatically detected during a 10 min test session. Beam interruption information was processed in the activity cage software to provide an index of horizontal movements. Rats were acclimatised for 1 h to the test room, before placing the animal in the activity cage (exposure) [25]. The basal activity counts of rats were pretested for a 15 min interval the day before the experiment to habituate them to the apparatus; they were adapted for 5 min and the basal activity counts were then recorded for 10 min [26].

### Systolic Blood Pressure (SBP) Measurement

Blood pressure was measured non-invasively on day 0 and 1 h following the last drug administration using tail-cuff technique attached to blood pressure recorder (UGO BASILE 58000, Italy).

### Urine and serum biochemical analysis

On day 2, urine samples were collected from animals of all groups through the housing in individual metabolic cages for 24 h for estimation of urinary total protein (UTP) using commercial reagent kit (Stanbio, USA). Blood samples were withdrawn via the retro-orbital plexus under ether anaesthesia from all rats on day 3, after 1 h of the last drug administration. The serum was isolated for estimation of blood urea nitrogen (BUN), serum creatinine (SCr), potassium (K^+^) and sodium (Na^+^) levels, using specific commercial kits, (Stanbio, USA), (Quimica Clinica Aplicada S.A., Spain), (Quimica Clinica Aplicada S.A., Spain), and (Teco Diagnostics, USA), respectively.

### Renal tissue biochemical and histopathological analysis

Directly after collecting the blood samples, rats were sacrificed by cervical dislocation under ether anaesthesia and both kidneys were isolated. The right kidneys were rinsed in chilled 0.9 % NaCl (pH 7.4) then homogenised. The homogenates were used for estimation of kidney contents of lipid peroxides measured as malondialdehyde (MDA) according to Ruiz-Larrea et al. [27], reduced glutathione (GSH) according to [40] and NOx (nitrite and nitrate, stable metabolites of NO) using commercial reagent kit (Cayman chemical company, Germany).

The left kidneys from all groups were removed and fixed in 10% neutral buffered formal saline for 72 h at least. All the specimens were washed in tap water for half an hour and then dehydrated in ascending grades of alcohol, cleared in xylene and embedded in soft paraffin. Paraffin sections of 5 μm thick were stained with haematoxylin and eosin (H&E) [28], for histopathological examination. Images were captured and processed using Adobe Photoshop version 8.0.

### Statistical Analysis

All the values are presented as means ± standard error of the means (SEM). Comparisons between different groups were carried out using one-way analysis of variance (ANOVA) followed by Tukey HSD test for multiple comparisons [29]. Graphpad Prism software, version 5 was used to carry out these statistical tests. For locomotor activity, square root transformed percent was calculated [30], while Statistica version 7 was used for two-way ANOVA followed by Tukey HSD as multiple comparison tests for blood pressure analysis. The difference was considered significant when *p* < 0.05.

## Results

### Locomotor activity of rats

Gly model of RM-induced ARF markedly decreased the basal locomotor activity on day 3 of Gly administration, compared with normal group. Pretreatment with Carv (2.5 mg/kg) and Nebi (10 mg/kg) led to a significant protection in locomotor activity on day 3 from Gly administration compared to ARF group (Table 1).

**Table 1:** Locomotor activity

Parameter Groups	Locomotor activity			

	Count/10 min		Percentage of basal activity	Square-root- transformed % of basal activity

	Day 0	Day 3	Day 3 / Day 0	Day 3
Saline	271.50 ± 8.08	199.33 ± 5.11	73%	0.96^b^± 0.02
Gly	172.10 ± 9.23	28.90 ± 2.19	17%	0.41^a^± 0.02
Gly-Carv	178.30 ± 8.27	116.90 ± 6.73	65%	0.81^ab^± 0.03
Gly-Nebi	187.80 ± 16.57	113.50 ± 7.14	60%	0.80^ab^± 0.04

Saline, rats treated with saline and considered as normal rats; Gly, rats treated with glycerol; Gly-Carv, rats treated with glycerol and carvedilol; Gly-Nebi, rats treated with glycerol and nebivolol. Data are presented as mean ± SE, n=10.

a Significantly different from Saline; *p* < 0.05.

b Significantly different from Gly; *p* < 0.05.

### Systolic blood pressure

**Figure 1:**
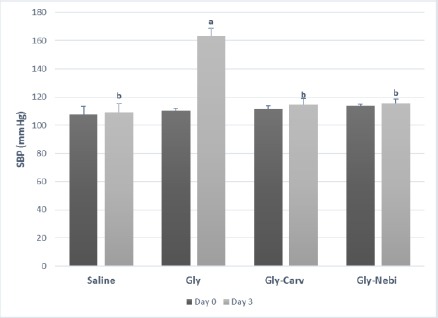
Systolic blood pressure. Saline, rats treated with saline and considered as normal rats; Gly, rats treated with glycerol; Gly-Carv, rats treated with glycerol and carvedilol; Gly-Nebi, rats treated with glycerol and nebivolol. Data are presented as mean ± SE, n=10. a Significantly different from Saline; p < 0.05. b Significantly different from Gly; p < 0.05

Gly markedly increased the basal SBP of rats on day 3. However, pretreatment of rats with Carv and Nebi significantly protected against this Gly-induced elevation of SBP (Fig. 1).

### Urine and serum biochemical analysis

Induction of ARF in rats by a single dose of Gly markedly increased the normal UTP on day 2 of Gly administration and increased SCr and BUN levels on day 3. A marked decrease in normal Na^+^ level and increase in K^+^ level were also observed on day 3. Pretreatment of rats with Carv and Nebi preserved the normal levels of UTP, SCr, BUN, Na^+^, and K^+^ (Table 2).

**Table 2:** Levels of urine total protein, serum creatinine, blood urea nitrogen, serum sodium and serum potassium

Parameters Groups	UTP (mg/dl)	SCr (mg/dl)	BUN (mg/dl)	Na^+^ (mEq/l)	K^+^ (mmol/l)
Saline	30.11^b^± 2.48	0.48^b^± 0.01	21.38^b^± 0.95	147.65^b^± 1.86	3.79^b^± 0.09
Gly	156.08^a^± 14.34	3.75^a^± 0.25	49.10^a^± 2.03	122.03^a^± 2.36	7.17^a^± 0.24
Gly-Carv	53.58^b^± 3.95	0.96^b^± 0.09	21.97^b^± 1.47	140.56^b^± 2.36	4.10^b^± 0.25
Gly-Nebi	53.14^b^± 3.73	1.06^b^± 0.09	22.14^b^± 1.41	140.63^b^± 2.72	4.19^b^± 0.17

Saline, rats treated with saline and considered as normal rats; Gly, rats treated with glycerol; Gly-Carv, rats treated with glycerol and carvedilol; Gly-Nebi, rats treated with glycerol and nebivolol; UTP, urine total protein; SCr, serum creatinine; BUN, blood urea nitrogen; Na^+^, serum sodium; K^+^, serum potassium. Data are presented as mean ± SE, n=10.

a Significantly different from Saline; *p* < 0.05.

b Significantly different from Gly; *p* < 0.05.

### Renal tissue biochemical analysis

**Figure 2:**
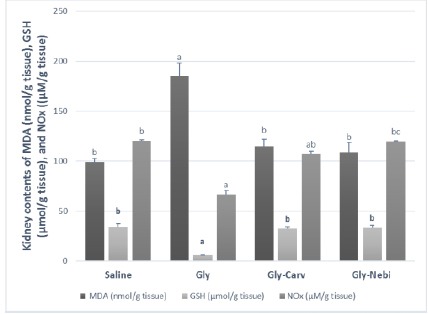
Kidney contents of malondialdehyde, reduced glutathione and nitric oxide. Saline, rats treated with saline and considered as normal rats; Gly, rats treated with glycerol; Gly-Carv, rats treated with glycerol and carvedilol; Gly-Nebi, rats treated with glycerol and nebivolol; MDA, malondialdehyde; GSH, reduced glutathione; NOx, nitrite and nitrate, stable metabolites of NO. Data are presented as mean ± SE, n=10. a Significantly different from Saline; p < 0.05. b Significantly different from Gly; p < 0.05. c Significantly different from Gly-Carv; p < 0.05

Induction of ARF in rats using Gly markedly increased the normal renal MDA level by 86% and decreased GSH and NOx levels by 83% and 44%, respectively. Pretreatment of rats with Carv or Nebi conserved the normal renal levels of MDA and GSH. Moreover, a marked protection of kidney NOx level was also detected; this protection was more significant in the group treated with Nebi rather than Carv (Fig. 2).

### Histopathological features of the renal tissues

**Figure 3:**
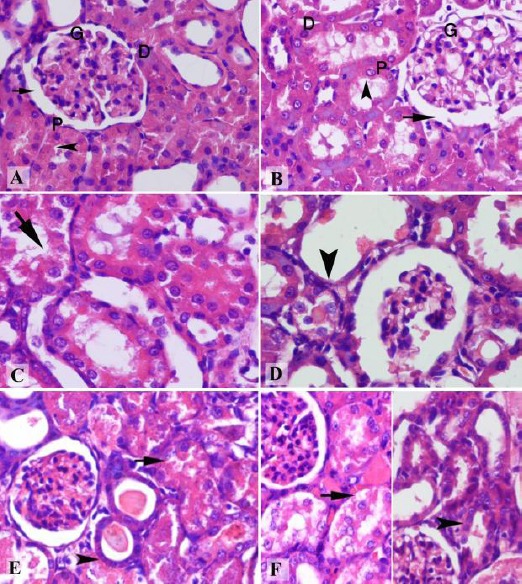
Histopathological features of the renal tissues. Photomicrographs of renal sections from rats treated with the following: Saline (A) & (B) show normal glomeruli (G) enclosed in Bowman’s capsule and separated from it by the urinary space (arrow), proximal convoluted tubules (P) with their brush borders (arrowhead), and distal convoluted tubules (D); Gly (C & D) show marked vacuolar degeneration in proximal tubules with discontinuity of the brush border (arrow), widening of urinary space, and a marked decrease in the height of the lining epithelium of distal tubules with widening of their lumen (arrowhead); Gly-Carv (E) shows a significant decrease in vacuolar degeneration in proximal tubules (arrow), an increase in the height of the lining epithelium of the distal tubules with no signs of vacuolar degeneration (arrowhead), and a normal glomeruli; Gly-Nebi (F) shows the persistence of the Gly-induced vacuolar degeneration, especially in proximal tubules, (arrow in the left part of the figure), less dilated lumens of distal convoluted tubules, and a normal urinary space (arrowhead in the right part of the figure). (H & E X 200)

The renal tissue of the normal rats showed normal glomeruli formed of a tuft of capillaries enclosed in Bowman’s capsule and separated from it by the urinary space. Two types of tubules were also observed, proximal convoluted tubules with their brush borders and distal convoluted tubules (Fig. 3. A & B). In the rat sacrificed 72 h following Gly administration, a marked vacuolar degeneration in proximal tubules with discontinuity of the brush border as well as a widening of urinary space of glomeruli were observed (Fig. 3. C). In addition, a marked decrease in the height of the lining epithelium of distal tubules and widening of their lumen are also noticed (Fig. 3. D). On the other hand, the renal tissues of a rat with Gly-induced ARF that were pretreated with Carv showed a significant decrease in vacuolar degeneration induced by Gly in proximal tubules, and the distal tubules showed an increase in the height of their lining epithelium with no signs of vacuolar degeneration. The glomeruli appeared more or less normal (Fig. 3. E). the renal sections of rats treated with Nebi-Gly showed the persistence of the Gly-induced vacuolar degeneration, especially in proximal tubules. However, the lumens of the distal convoluted tubules were less dilated if compared to the Gly-treated group, while the urinary space appeared more or less normal (Fig. 3. F).

## Discussion

Induction of RM-mimicking ARF in rats, in the current study, with Gly that was evidenced by the impairment of the kidney function biomarkers and confirmed by the histopathological findings is in accordance with other studies reported that the acute volume depletion model of Gly-ARF induces a closely related syndrome to the RM-ARF in human beings [31]. Renal vasoconstriction and hypoperfusion have been suspected to play a major role in the pathogenesis of this model [32].

Several potential mechanisms may contribute to this renal vasoconstriction. Muscle necrosis creates a dramatic fluid third spacing, leading to intravascular volume depletion and hypotension; this impairs the renal perfusion and causes a severe renal ischemia and tubular dysfunction [4]. The decreased serum Na^+^ level observed in the current Gly model of RM-ARF, and before [33], indicated this tubular dysfunction with a decreased Na^+^ reabsorption. However, a significant increase in serum Na^+^ levels was reported in other studies [34, 35]. This may be due to acute tubular necrosis that could lead to a decrease in the number of functioning nephrons. This effect may trigger multiple adaptive processes in the hyper-functioning remaining nephrons, most notably the augmented rates of Na^+^ reabsorption that lead to hypernatremia. On the other hand, the observed increase in the serum K^+^ level in the present Gly model of RM-ARF, which is correspondingly reported previously [33, 36], could be explained by the direct release of the intracellular K^+^ from the damaged muscles [37]. Remarkably, this hyperkalemia has not been observed in other studies using different models of ARF rather than RM-ARF [35, 38]. The pathogenesis of Gly-induced RM-ARF can also involve Mb release from the damaged muscles that facilitates the production of reactive oxygen species (ROS) [10]. Oxidative stress has been found to cause renal damage [39]. It promotes the formation of a variety of vasoactive mediators that can affect the renal function directly by causing renal vasoconstriction and thus reduce the GFR [40]. In the present study, induction of renal oxidative stress by Gly was demonstrated clearly by a significant increase in the normal renal MDA and decrease in GSH contents. A similar pattern was recorded by many studies [41, 42]. The increased tissue levels of ROS can also oxidize the locally released NO and diminishes its bioactivity [43]. Correspondingly, a significant decrease in the normal renal tissue content of NOx was demonstrated in the present Gly-ARF model, a result that is in line with other studies [44, 45].

The current Gly-induced RM-ARF was accompanied with a significant decrease in the normal locomotor activity of rats. It has been found that renal failure results in an accumulation of numerous organic substances that possibly act as neurotoxins and result in a development of a case that is known as uremic encephalopathy [46]. Uremic encephalopathy is associated with a generalised decrease in brain energy use, and thus a decrease in the locomotor activity [46].

Moreover, a significant increase in the SBP was also observed in the current Gly-ARF model. A similar finding was observed with gentamicin-induced ARF model [47]. Renal failure reduces the afferent glomerular arteriolar pressure, leading to the activation of the renin-angiotensin system, leading to hypertension [48]. Co-treatment of rats with either Carv or Nebi showed a significant protective effect against the current Gly-induced RM-ARF model. This observed renoprotective effect is in agreement with the findings of studies that used Carv or Nebi as a protective agent against other models of ARF in which restoration of the normal levels of renal function biomarkers was reported [49, 50].

The significant attenuation of Gly-induced oxidative stress in the rats treated with Carv or Nebi indicates that antioxidant pathway played a role in the renoprotective effects of both drugs. Many studies also reported this antioxidant effect of Carv and Nebi [49, 51]. Carv has been found to scavenge oxygen radicals and inhibit their release from activated neutrophils [52, 53]. It was found to accumulate in specific plasma membrane sites allowing it to approach the site of fatty acid side chain unsaturation where lipid peroxidation is thought to occur; this explains its high potency as an antioxidant [54]. On the other hand, Nebi has vasodilating properties mediated by direct stimulation of eNOS, thereby increasing the availability of NO [19]. It has been shown that NO donors can scavenge ROS by the NADPH oxidase [55].

The significant improvement of the serum Na^+^ and K^+^ levels observed in Gly-Carv and Gly-Nebi as compared to Gly group indicated a protective effect of Carv and Nebi against Gly-induced hyponatremia and hyperkalemia. In addition, adrenergic β-blockade would increase proximal Na^+^ reabsorption [56], contributing to the drugs-induced hypernatremia. Correspondingly, in the previous study, Nebi, in combination with hydrochlorothiazide, reduced the Na^+^ clearance [57]. On the contrary, Rodriguez-Perez *et al*. [58], and Greven and Gabriels [59] reported that Carv and Nebi, respectively, produced a significant natriuresis followed by hyponatremia in rats with severe nephrosclerosis. This natriuresis was attributed to a compensatory renal mechanism due to an improvement of GFR produced by those drugs, which in turn increased urinary excretion of Na^+^ and fluids. On the other hand, the antioxidant activities of Carv and Nebi could explain the reversal of ROS-induced hyperkalemia that resulted from the loss of intracellular K^+^ due to the increasing cell membrane permeability by membrane lipids peroxidation [60, 61]. In addition, Carv by having a α_1_-adrenergic blocking activity can retain K^+^ intracellularly, contributing to hypokalemia induced by Carv [62]. On the contrary, it has been suggested that β-adrenergic receptor antagonism could suppress the renin-angiotensin aldosterone system (RAAS), by inhibiting renin secretion, hence, predisposing patients to K^+^ retention [63].

In addition to those observed renoprotective effects of Carv and Nebi that consequently caused an improvement of the locomotor activity of rats, both have been reported to have a direct neuroprotective effect [64-66]. Carv protected against 3-nitropropionic acid induced behavioural alterations in rats [67], and Nebi improved the neurological status and the hind limb motor function in a spinal cord ischemia/reperfusion injury model in rabbits [65, 66]. Therefore, this improvement in the locomotor activity demonstrated in the current study could be up to a point due to a direct neuroprotective effect against uremic encephalopathy. Similarly, the protective effect of Carv and Nebi against Gly-induced SBP-elevation could be accounted partly for the observed renoprotective effect of those treatments and also to their renowned direct antihypertensive effects [68, 69]

The present data revealed that animals treated with Carv or Nebi showed a significant increase in renal NOx content as compared to Gly group; this protection was more significant in the group treated with Nebi rather than Carv. Correspondingly, previous studies demonstrated that Carv and Nebi increased NO content [70-74].

Carv effects have been found to be blocked by the inhibition of eNOS enzyme using L-NAME [70]. This suggests that Carv’s actions are largely NO-mediated. Moreover, this might explain the current observed Carv-induced rise in renal NO content and suggests it to be dependent on stimulation of intact eNOS. The Nebi-induced elevation of NO content was more significant than that of Carv because Nebi can increase NO bioavailability by, at least, two mechanisms: by increasing NOS activity [75], or, under conditions of oxidative stress, by reducing O_2_^-^ generation and inhibiting eNOS uncoupling and, therefore, NO inactivation [76].

NO exerts a protective role against renal damage in several animal models of kidney disease as well as in human chronic renal failure. It promotes the increase of RBF and exerts antigrowth and antiproliferative effects on vascular smooth muscle [77]. It also plays an important role in regulating renal hemodynamic and functions [78]. Interestingly, Maree *et al*. [79] indicated that NOS inhibition worsens Gly-induced ARF model, while NO supplementation protects against it.

In conclusion, the present study revealed that treatment of rats with Carv (2.5 mg/kg/day, po) or Nebi (10 mg/kg, po) protected against renal damage involved in Gly-induced RM-mimicking ARF. The findings demonstrated the involvement of the antioxidant and NO releasing properties of both drugs and suggested their involvement in this renoprotective effect.
